# Inadequate sleep increases stroke risk: evidence from a comprehensive meta-analysis of incidence and mortality

**DOI:** 10.1007/s11357-025-01593-x

**Published:** 2025-03-12

**Authors:** Zoltan Ungvari, Mónika Fekete, Andrea Lehoczki, Gyöngyi Munkácsy, János Tibor Fekete, Virág Zábó, György Purebl, Péter Varga, Anna Ungvari, Balázs Győrffy

**Affiliations:** 1 Vascular Cognitive Impairment, Neurodegeneration and Healthy Brain Aging Program, Department of Neurosurgery, University of Oklahoma Health Sciences Center, Oklahoma City, OK USA; 2 Stephenson Cancer Center, University of Oklahoma, Oklahoma City, OK USA; 3 Oklahoma Center for Geroscience and Healthy Brain Aging, University of Oklahoma Health Sciences Center, Oklahoma City, OK USA; 4 Department of Health Promotion Sciences, College of Public Health, University of Oklahoma Health Sciences Center, Oklahoma City, OK USA; 5 International Training Program in Geroscience, Doctoral College, Health Sciences Division/Institute of Preventive Medicine and Public Health, Semmelweis University, Budapest, Hungary; 6 Institute of Preventive Medicine and Public Health, Semmelweis University, Semmelweis University, Budapest, Hungary; 7 Doctoral College, Health Sciences Division, Semmelweis University, Budapest, Hungary; 8 Dept. Of Bioinformatics, Semmelweis University, 1094 Budapest, Hungary; 9 Cancer Biomarker Research Group, Institute of Molecular Life Sciences, HUN-REN Research Centre for Natural Sciences, 1117 Budapest, Hungary; 10 Institute of Behavioural Sciences, Semmelweis University, Budapest, Hungary; 11 Dept. Of Biophysics, Medical School, University of Pecs, 7624 Pecs, Hungary

**Keywords:** Healthy aging, Circadian, Unhealthy aging, Sleep duration, Stroke, Incidence, Mortality, Risk factor, Cohort studies, Shift workers

## Abstract

The link between abnormal sleep duration and stroke outcomes remains contentious. This meta-analysis quantifies how both short and long sleep durations impact stroke incidence and mortality. A comprehensive search was conducted in PubMed, Web of Science, Cochrane Library, Embase, and Google Scholar up to November 1, 2024, to identify cohort studies evaluating sleep duration and stroke outcomes. Meta-analysis was performed using MetaAnalysisOnline.com and a random-effects model to estimate pooled hazard ratios (HRs). Results were visualized through Forest and Funnel plots. Analysis of 43 studies (35 on stroke incidence, 8 on mortality) revealed significant associations between sleep duration and stroke outcomes. Short sleep duration (≤ 5–6 h) was associated with increased stroke incidence (HR 1.29, 95% CI 1.19–1.40, *p* < 0.01) and modestly elevated mortality (HR 1.12, 95% CI 1.01–1.25, *p* = 0.03). Long sleep duration (> 8–9 h) demonstrated stronger associations with both increased stroke incidence (HR 1.46, 95% CI 1.33–1.60, *p* < 0.01) and mortality (HR 1.45, 95% CI 1.31–1.60, *p* < 0.01). Significant heterogeneity was observed in incidence studies (*I*2 = 74–75%), while mortality analyses showed moderate to low heterogeneity (*I*2 = 35–40%). This meta-analysis highlights a U-shaped association between sleep duration and stroke risk, with both short and long sleep durations linked to higher stroke incidence and mortality. These findings underscore the importance of balanced sleep duration as a modifiable risk factor in stroke prevention strategies and provide a foundation for the Semmelweis Study, a prospective workplace cohort investigating the role of modifiable lifestyle factors in unhealthy cerebrovascular and brain aging.

## Introduction

Sleep is a fundamental determinant of health, influencing numerous physiological systems [1–9] and contributing significantly to the risk of major chronic, age-related diseases [10–19]. Among these, the cerebrovascular system is particularly vulnerable to disruptions in sleep patterns [16, 20, 21]. Stroke, a leading cause of mortality and long-term disability worldwide, has emerged as a focal point in research examining the interplay between sleep and disease risk [5, 22–27]. While both short and long sleep durations have been linked to an increased risk of cardiovascular events, their precise contributions to stroke incidence and mortality remain incompletely understood.

Modern societal and occupational demands have led to widespread disruptions in sleep patterns. Irregular work schedules, shift work, pervasive workplace stress, and the ubiquitous use of digital devices are significant contributors to sleep disturbances [28–31], making insomnia one of the most prevalent medical complaints of the twenty-first century [32–34]. Healthcare professionals, such as doctors and nurses, are particularly vulnerable due to the irregular schedules imposed by shift work and high job demands [35–38]. Digital device usage exacerbates sleep disturbances by misaligning sleep onset and disrupting circadian rhythms through blue light exposure, while work-related stress contributes to insomnia and reduced sleep quality [39]. These interconnected factors underscore the complex, multifactorial nature of sleep disturbances in contemporary society, particularly in high work demand professions.

Emerging evidence highlights the need to incorporate sleep as a key variable in population-based health research [6, 7, 19, 40, 41] and as an important highlight of health promotion programmes. The Semmelweis Study, a newly launched prospective workplace cohort at one of Central Europe’s largest health sciences universities, aims to address this need [42]. The study focuses on identifying the determinants of unhealthy aging, with particular attention to modifiable lifestyle factors [43, 44], including sleep and circadian rhythm. With a cohort enriched by shift workers, the Semmelweis Study is uniquely positioned to explore how occupational and lifestyle factors influence sleep patterns and to investigate how circadian misalignment and irregular sleep patterns amplify the risk of stroke and other age-related diseases. By leveraging longitudinal data and employing both subjective and objective measures of sleep, the study will comprehensively evaluate not only sleep duration but also quality, timing, and the broader psychosocial and environmental determinants of sleep health. The results of this study may help to design and implement workplace-based intervention programmes that can effectively reduce the increased risk of irregular work.

Building on the framework of our prior meta-analysis linking sleep duration to all-cause mortality [45], this study synthesizes data from diverse populations to explore the association between sleep patterns and stroke risk. Importantly, the findings of this meta-analysis provide critical insights that will inform the design and analytic strategies of the Semmelweis Study. These findings underscore the importance of optimizing sleep as a key preventive strategy for reducing stroke risk and overall morbidity, and also lay the groundwork for future research to develop targeted interventions and public health strategies that address this critical risk factor in shift workers and the broader population.

## Methods

### Search strategy and selection criteria

We carried out a systematic review and meta-analysis to investigate the association between sleep duration and both stroke incidence and stroke-related mortality. For this purpose, we searched several electronic databases, including PubMed, Web of Science, the Cochrane Library, Embase, and Google Scholar, to identify relevant studies. To ensure a thorough exploration of the literature, we employed various keyword combinations, including but not limited to “sleep duration AND stroke,” “short sleep AND stroke,” “long sleep AND stroke,” “sleep AND mortality,” and “sleep duration AND stroke mortality.” This comprehensive approach aimed to capture all relevant studies examining the influence of sleep duration on stroke risk and stroke-related death. Most studies categorized 7–8 h of nightly sleep as the reference or “normal” duration, with “short” sleep defined as ≤ 5–6 h and “long” sleep as > 8–9 h. Only studies published in English were included. We also collected studies from previous metaanalyses [5, 22–27].

Eligibility assessment was conducted by two independent reviewers who screened articles against predefined inclusion and exclusion criteria. To be included in the meta-analysis, studies had to meet the following criteria: they examined the relationship between sleep duration and either stroke incidence or stroke mortality; diagnosed strokes according to international standards; adopted a longitudinal study design; measured sleep duration through self-report, questionnaires, clinical evaluations, or objective methods; and provided association measures such as odds ratios (ORs), relative risks (RRs), or hazard ratios (HRs). We excluded case reports, conference abstracts, and commentary pieces.

The inclusion criteria were as follows: (1) studies investigating the link between sleep duration and stroke incidence or mortality; (2) stroke diagnoses consistent with international guidelines; (3) longitudinal design; (4) sleep duration assessed through self-reports, questionnaires, clinical evaluations, or objective tools; and (5) provision of ORs, RRs, or HRs with sufficient adjustment for confounders. Exclusion criteria encompassed (1) non-empirical studies, such as case reports or conference abstracts; (2) studies not addressing the specific relationship between sleep duration and stroke outcomes; (3) cross-sectional designs; (4) stroke diagnoses lacking sufficient detail; and (5) studies without relevant association metrics.

**Fig. 1:**
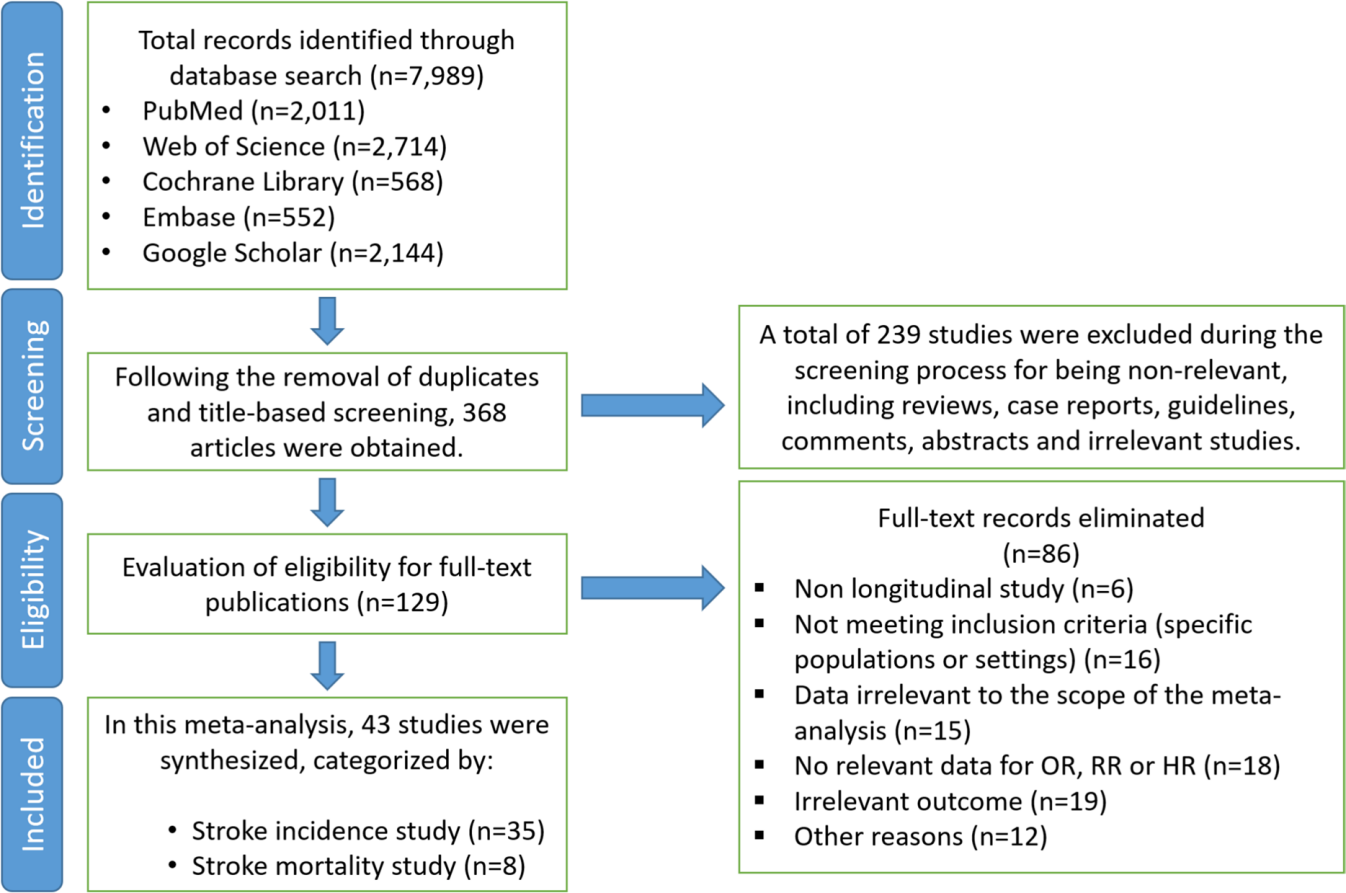
Flow diagram illustrating the study selection process for the meta-analysis on the association between sleep duration and stroke incidence and mortality. A total of 7989 records were identified through database searches. After removing duplicates and irrelevant articles 43 studies were included in the meta-analysis, categorized as stroke incidence studies (*n* = 35) and stroke mortality studies (*n* = 8)

Extracted information included the first author’s name, publication year, study design, total sample size, and the number of stroke cases or deaths reported during the follow-up period. Where multiple levels of adjustment were reported, we utilized data from the most fully adjusted model. Details of the selection process are presented in Fig. 1.

### Statistical analysis

All statistical analyses were conducted using the web-based platform MetaAnalysisOnline.com. Pooled risk estimates, such as hazard ratios (HRs) and their corresponding 95% confidence intervals (CIs), were calculated using a random-effects model. This model accommodates variability between studies, thereby improving the generalizability of the findings. To facilitate interpretation and highlight heterogeneity, forest plots were created to display the results of individual studies alongside the overall pooled estimate.

Heterogeneity among the included studies was evaluated using Cochran’s *Q* test and the *I*2 statistic. Cochran’s *Q* test, based on a chi-square distribution, was applied to determine whether the observed variation in effect sizes exceeded what could be attributed to chance alone. The *I*2 statistic quantified the percentage of total variability that arose from true differences between studies rather than random sampling error.

### Assessment of publication bias

Potential bias in the published literature was examined through both graphical and statistical methods. Funnel plots, which plot effect sizes against measures of study precision, were visually inspected for asymmetry, an indicator of publication bias. Additionally, Egger’s regression analysis was conducted to quantitatively assess the relationship between effect sizes and their standard errors, providing further insight into potential bias.

### Subgroup analyses

Subgroup analyses were conducted separately for stroke incidence and stroke-related mortality. For each subgroup, we calculated pooled effect estimates and assessed heterogeneity using the same statistical methods applied to the overall analysis. In some cases, data were available only for male or female cohorts. Due to the limited number of such studies, separate subgroup analyses for these populations were not performed. Instead, combined cohort data were used to derive aggregated effect estimates across all included participants.

## Results

### Association between short sleep and stroke incidence

**Fig. 2:**
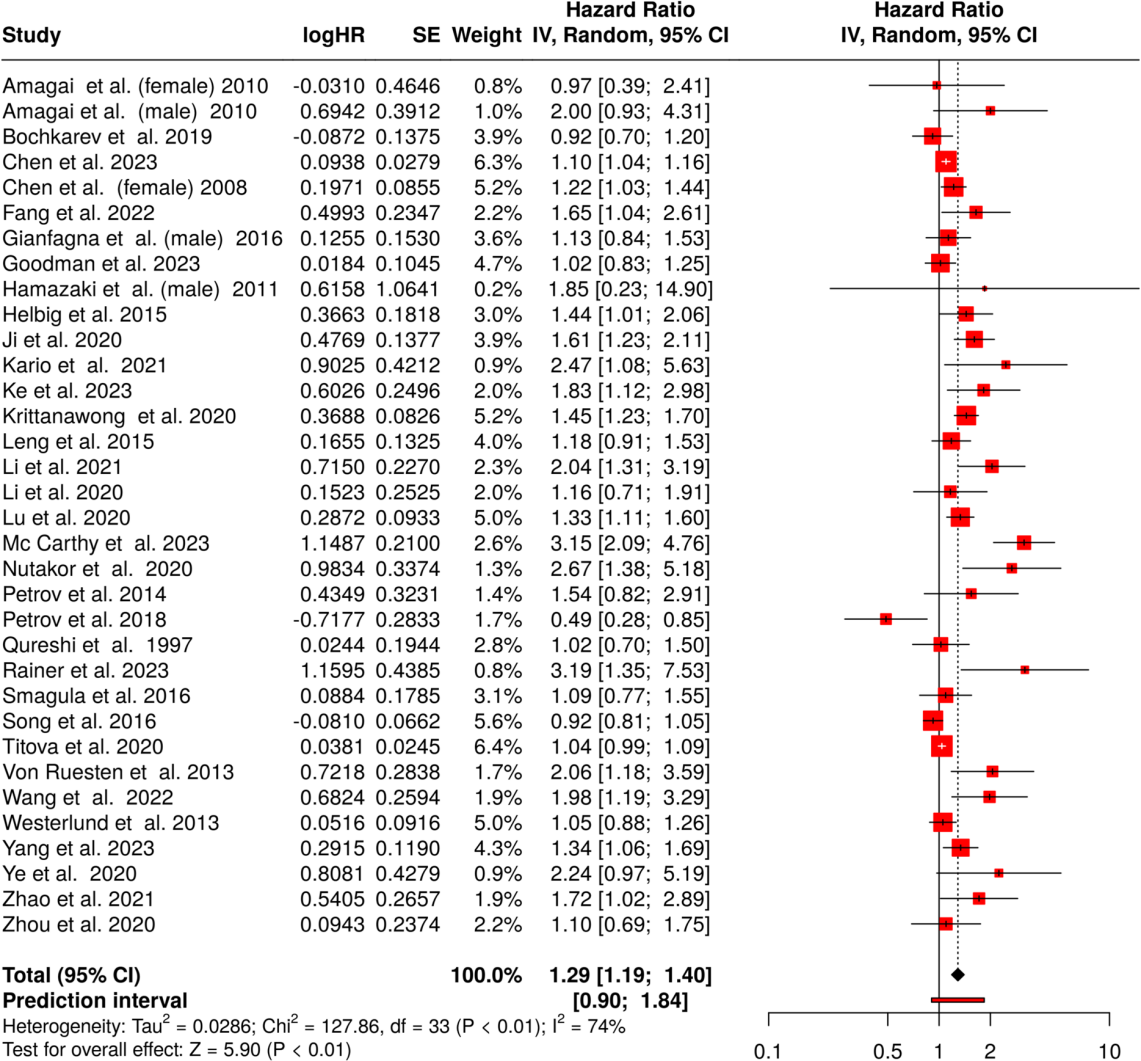
Forest plot showing the association between short sleep duration and the incidence of stroke. Hazard ratios (HRs) with 95% confidence intervals (CIs) are presented for individual cohorts. The size of the squares reflects the weight of each study in the meta-analysis, and the diamond represents the pooled estimate. The overall HR indicates an increased risk of stroke associated with short sleep duration (HR 1.29, 95% CI 1.19–1.40). The analysis demonstrates high heterogeneity (*I*2 = 74%, *p* < 0.01) across studies. Abbreviations: CI, confidence interval; HR, hazard ratio; IV, inverse variance; SE, standard error

The meta-analysis evaluating the impact of short sleep duration on stroke incidence encompassed data from 34 cohorts (Fig. 2) [46–80]. Employing random effects model with inverse variance methodology, we identified a pooled HR of 1.29 (95% CI 1.19–1.40), indicating a statistically significant association (*p* < 0.01) between short sleep and elevated stroke risk.

However, the analysis identified significant between-study heterogeneity (*p* < 0.01). The *I*2 statistic of 74% indicates a high-degree of variability, suggesting that the most of the differences in effect estimates across studies stem from variations in study design, methodology, or population characteristics, rather than random fluctuation.

**Fig. 3:**
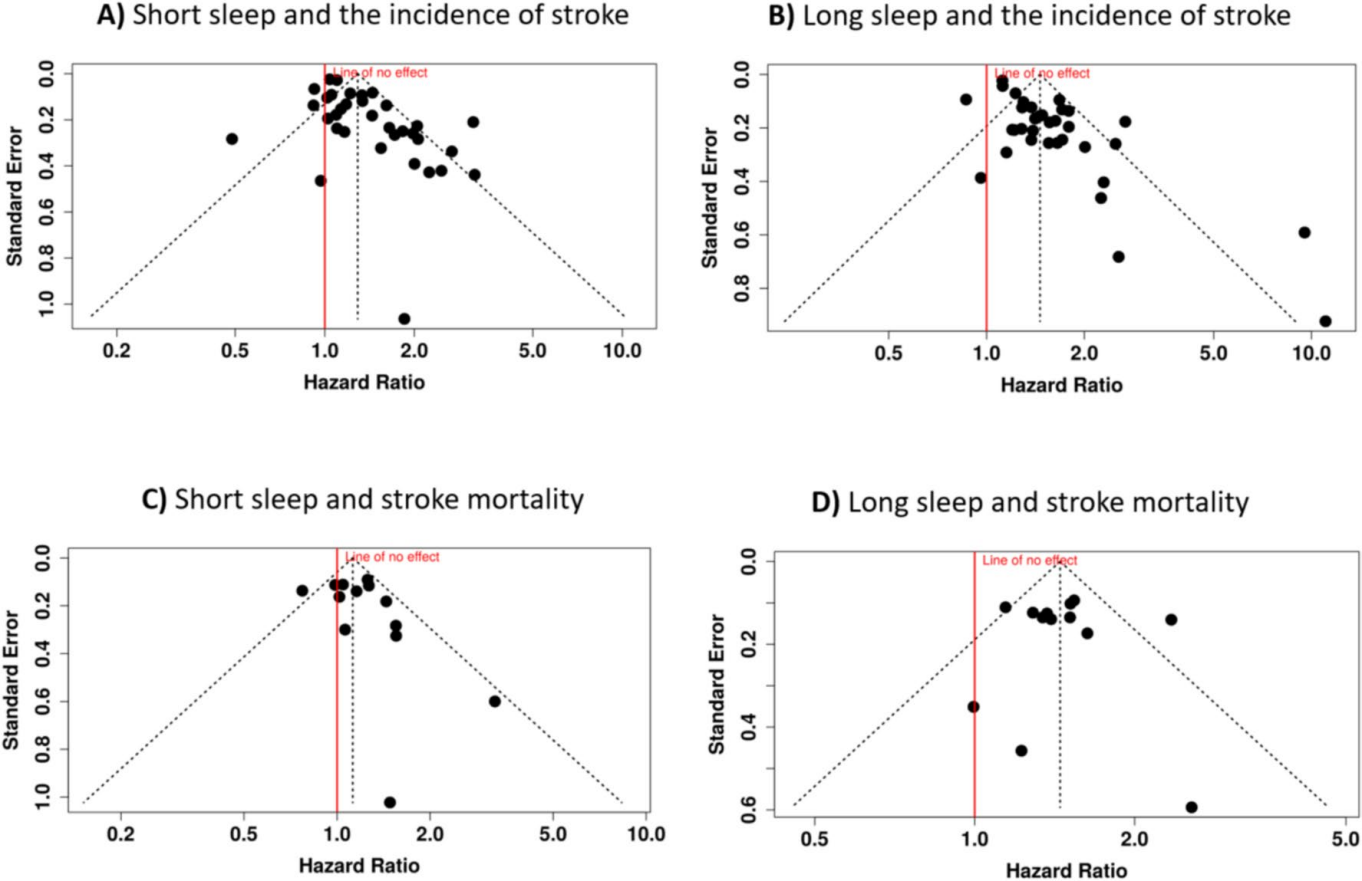
Funnel plots depicting the relationship between hazard ratios and standard errors for the association between sleep duration and stroke events. Plots are stratified by sleep duration as short sleep (**A**, **C**) or long sleep (**B**, **D**) and stroke outcomes as incidence (**A**, **B**) or mortality (**C**, **D**). Each dot represents a single cohort included in the meta-analysis. Funnel plot asymmetry, indicated by deviations from symmetry around the vertical reference line, suggests potential for publication bias in **A** and **B**, suggesting that studies with smaller or non-significant effects may be underrepresented

Further investigation using funnel plot (Fig. 3A) exhibited asymmetry, and Egger’s test provided statistical verification of a potential publication bias (intercept, 1.52; 95% CI 0.78–2.26; *t* = 4.03, *p* < 0.01). This finding suggests that the available evidence may not fully represent the complete spectrum of research on this topic, potentially leading to an overestimation of the effect size.

### Association between long sleep and stroke incidence

**Fig. 4:**
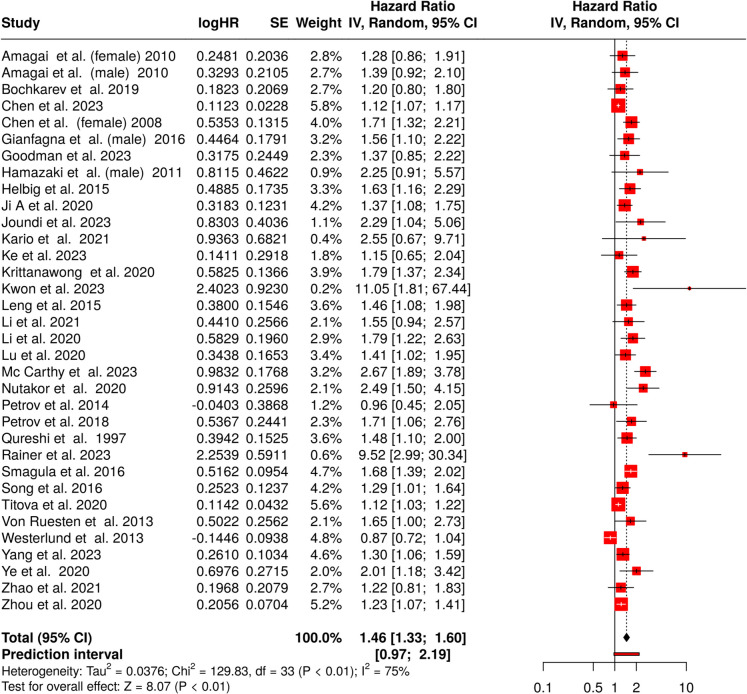
Forest plot summarizing the association between long sleep and stroke incidence. The meta-analysis shows a significant pooled hazard ratio (HR 1.46, 95% CI 1.33–1.60), indicating increased stroke risk with long sleep. High heterogeneity (*I*2 = 75%, *p* < 0.01) was observed across studies. Abbreviations: CI, confidence interval; HR, hazard ratio; IV, inverse variance; SE, standard error

The meta-analysis investigating the impact of long sleep duration on stroke incidence incorporated data from 34 cohorts (Fig. 4) [46–80]. Employing random effects model with inverse variance methodology, we identified a statistically significant pooled HR of 1.46 (95% CI 1.33–1.60), indicating a significantly elevated stroke risk associated with prolonged sleep. However, the analysis revealed substantial between-study heterogeneity (*p* < 0.01). The *I*2 statistic of 75% indicates significant variability among the included studies, suggesting that differences in effect sizes across cohorts are likely due to actual differences in study characteristics or populations, rather than random chance. This reflects variation in the magnitude and potentially in the direction of the effects.

The funnel plot (Fig. 3B) displayed potential asymmetry, and the Egger’s test provided statistical confirmation of a publication bias (intercept, 1.91; 95% CI 1.26–2.56; *t* = 5.779; *p* < 0.01). This finding suggests that the available evidence may not fully represent the complete spectrum of research on this topic, potentially leading to an overestimation of the effect size.

### Association between short sleep and stroke mortality

**Fig. 5:**
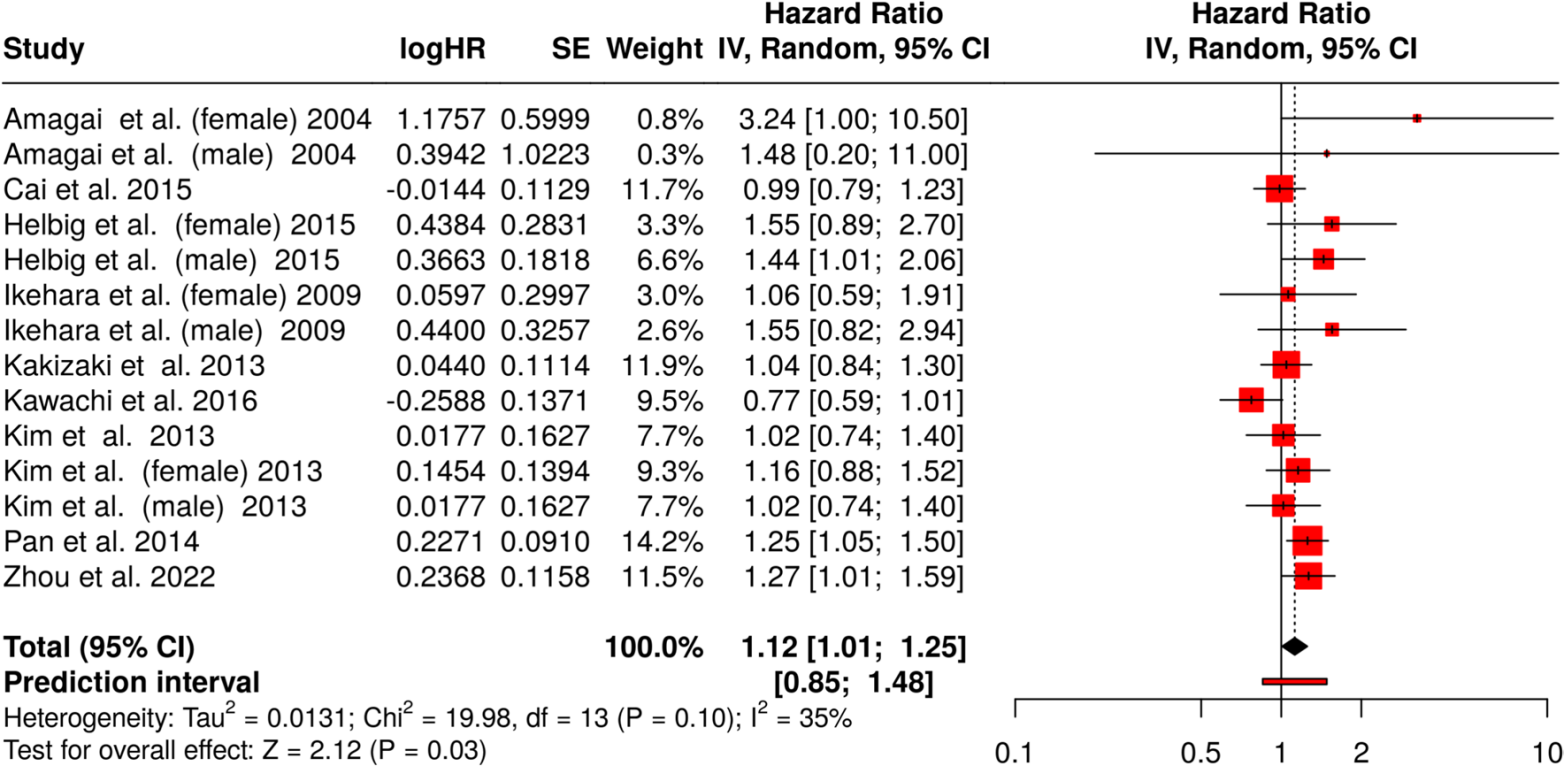
Forest plots of hazard ratios (HR) for the association between short sleep duration and mortality risk from stroke, based on a meta-analysis of observational cohorts (*n* = 14). The HRs and their 95% confidence intervals (CI) are displayed, providing a visual summary of the magnitude and precision of the effects across the different study populations. The combined HR is 1.12 (95% CI 1.01, 1.25), indicating a 12% higher mortality risk with short sleep. Abbreviations: CI, confidence interval; HR, hazard ratio; IV, inverse variance; SE, standard error

In this meta-analysis examining the association between short sleep duration and stroke mortality, a total of 14 trials [81–88] were included, representing both male and female cohorts (Fig. 5) as in the other analyses. The overall effect estimate was 1.12, with a 95% CI ranging from 1.01 to 1.25, and the result was statistically significant (*p* = 0.03). This finding suggests that individuals reporting shorter sleep duration have a slightly higher likelihood of death from stroke over the study period.

Heterogeneity across studies was relatively low, as indicated by a heterogeneity measure of 35%, suggesting that the differences in effect sizes between studies were not substantial. This consistency supports the robustness of the pooled estimate. The prediction interval, which provides an estimate of the range within which the true effect of short sleep duration on mortality might lie in future studies, ranged from 0.85 to 1.48. This range indicates some uncertainty about the generalizability of the findings to all populations and contexts, as the lower bound crosses the null value.

The evaluation of publication bias in this analysis was conducted using a funnel plot and Egger’s test for asymmetry. The funnel plot appeared symmetrical, suggesting the absence of substantial publication bias (Fig. 3C). This visual assessment was further supported by the results of Egger’s test, which did not detect significant asymmetry (intercept = 0.87, 95% CI − 0.57 to 2.32, *t* = 1.185, *p* = 0.259).

### Association between long sleep and stroke mortality

**Fig. 6:**
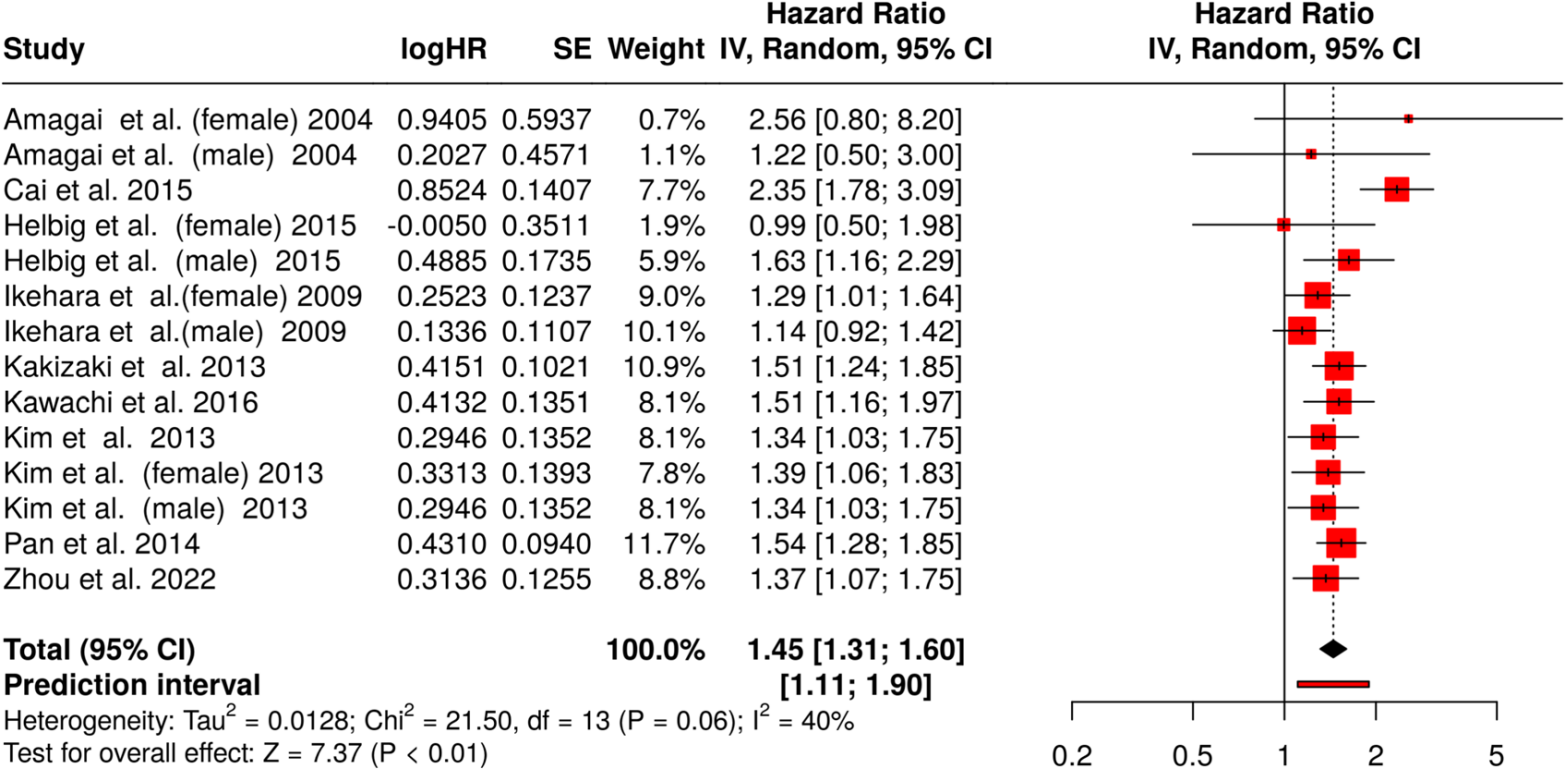
Forest plot showing the association between long sleep duration and stroke mortality (*n* = 14). The meta-analysis demonstrates a significant pooled hazard ratio (HR 1.45, 95% CI 1.31–1.60), indicating higher stroke mortality risk with long sleep. Moderate heterogeneity (*I*2 = 40%, *p* = 0.06) is observed across studies. Abbreviations: CI, confidence interval; HR, hazard ratio; IV, inverse variance; SE, standard error

For long sleep duration and stroke mortality, data from 14 cohorts were analyzed (Fig. 6) [81–88] (Fig. 6). The analysis was conducted using random-effects model with inverse variance method to estimate the pooled HR for long sleep duration concerning mortality risk. The pooled HR was 1.45, with a CI ranging from 1.31 to 1.60, indicating a 45% higher risk of mortality among individuals with prolonged sleep durations compared to those with normal sleep durations. This result was statistically significant (*p* < 0.01).

Despite the consistent direction of the findings, heterogeneity across studies was moderate. The heterogeneity analysis revealed a *p*-value of 0.06, and 40% of the observed variability (*I* [2]) in effect estimates was attributed to differences between studies rather than random chance. This level of heterogeneity suggests that variations in study design, population characteristics, or other contextual factors may contribute to differences in the strength of the observed association. Potential sources of heterogeneity include differences in sleep assessment methods (e.g., self-reported vs. objective), demographic characteristics, and geographic regions. Additionally, the definitions of short and long sleep duration varied across the included studies, with short sleep thresholds ranging from ≤ 6 to ≤ 7 h and long sleep thresholds ranging from ≥ 8 to ≥ 9 h. This inconsistency likely contributed to the observed heterogeneity in the pooled hazard ratios. Establishing standardized definitions in future studies would facilitate better comparability and enhance the reliability of meta-analytic estimates.

Assessment of publication bias was performed using a funnel plot and Egger’s regression test for asymmetry. The funnel plot appeared symmetrical, indicating no clear evidence of publication bias among the included studies. This visual observation was further confirmed by Egger’s test, which showed no statistically significant asymmetry. The intercept for Egger’s test was estimated at 0.14, with a CI ranging from − 1.78 to 2.07, and a *p*-value of 0.887, suggesting that the results were consistent with the absence of small-study effects or selective reporting.

## Discussion

The findings of this meta-analysis provide compelling evidence for a U-shaped relationship between sleep duration and stroke risk, with both short and long sleep durations associated with elevated risk for stroke incidence [46–80] and mortality [81–88]. These results reinforce the growing consensus that sleep and circadian rhythm are critical, modifiable determinants of cerebrovascular health, emphasizing the need for targeted interventions to optimize sleep duration as part of comprehensive stroke prevention strategies.

The association between short sleep duration and stroke risk can be attributed to several well-established mechanisms. Insufficient sleep (< 6 h) is increasingly recognized as a risk factor for cardiovascular and cerebrovascular diseases, likely mediated by mechanisms such as increased inflammation [12, 16, 89, 90], endothelial dysfunction [91–94], heightened blood pressure [95–97], and metabolic dysregulation [98–101]. Short sleep duration is associated with heightened sympathetic nervous system activation [97, 102], leading to elevated blood pressure and increased vascular tone, both of which exacerbate vascular stress and contribute to endothelial damage. Additionally, sleep deprivation disrupts the hypothalamic–pituitary–adrenal axis, resulting in elevated cortisol levels and an upregulation of pro-inflammatory mediators [103–108], such as IL-6 and TNF-α. This inflammatory state creates an environment conducive to atherosclerosis, thrombosis, and overall vascular dysfunction, increasing the likelihood of cerebrovascular events. Furthermore, chronic sleep deprivation impairs glymphatic system function, which is critical for the clearance of neurotoxic proteins, such as amyloid-β [109]. This impairment may contribute not only to neurovascular pathology but also to neurodegenerative processes that exacerbate stroke risk and compromise brain health [17, 110–113]. These findings reinforce the importance of promoting optimal sleep duration (7–8 h per night) as part of comprehensive public health strategies to reduce stroke risk. This nuanced understanding is essential for designing interventions tailored to high-risk populations, including those with pre-existing cardiovascular conditions or poor sleep hygiene.

In contrast, prolonged sleep duration (> 8–9 h) may reflect different sets of pathophysiological mechanisms, which often overlap with underlying health conditions. Long sleep duration is frequently associated with systemic inflammation and elevated C-reactive protein levels, contributing to a pro-inflammatory state that damages the endothelium and promotes thrombogenesis. Additionally, prolonged sleep may reflect subclinical disease, frailty, or neurodegenerative processes, all of which heighten vulnerability to cerebrovascular events. The phenomenon of long total sleep time can also be a sign of fragmented sleep architecture and/or poor-quality sleep where the total amount of key physiological variables (e.g., slow wave sleep and/or REM phases) is as small as in short sleep. Conditions such as heart failure, depression, and obstructive sleep apnoea, which are more common in individuals with prolonged sleep, may further amplify stroke risk. Age and sex [6, 90] may modulate the relationship between sleep duration and stroke risk. In older individuals, age-related changes in sleep architecture, including decreased slow-wave sleep and circadian misalignment, exacerbate vascular vulnerability and reduce resilience to cellular stress and inflammation. Similarly, older individuals may be more susceptible to the deleterious effects of abnormal sleep due to age-related impairment in cellular stress resilience pathways, vascular alterations, and a higher prevalence of coexisting conditions. Sex-specific differences, particularly hormonal influences such as estrogen [97, 106], may also play a role in modulating sleep duration, cardiovascular health, and stroke risk, warranting further investigation into sex-specific effects.

The findings of this meta-analysis have significant implications for the ongoing Semmelweis Study, a prospective workplace cohort based in a health sciences university setting [42]. By incorporating detailed assessments of sleep duration, quality, and circadian rhythms, the Semmelweis Study is uniquely positioned to address the role of sleep disturbances and occupational factors in healthy cerebrovascular and brain aging. Healthcare professionals, such as doctors and nurses, are particularly vulnerable to circadian misalignment and sleep disruption due to shift work and demanding schedules. Shift work is known to disrupt circadian rhythms, impair sleep duration and quality, and contribute to adverse metabolic and vascular outcomes, creating a substantial risk for cerebrovascular disease in this population. The Semmelweis Study provides a unique opportunity to examine these relationships in a cohort enriched with shift workers, offering valuable insights into the specific pathways through which occupational stress and sleep disruption contribute to stroke risk [42]. By incorporating both subjective (e.g., sleep questionnaires) and objective measures (e.g., actigraphy and wearable devices) of sleep duration and quality, the Semmelweis Study can generate high-quality data to address critical knowledge gaps. The longitudinal design of the study will allow for the examination of sleep as a continuous variable, facilitating the identification of dose–response relationships and thresholds for stroke risk. Additionally, the study’s ability to integrate demographic, occupational, and psychosocial factors will enable a deeper understanding of how sleep disturbances interact with other determinants of cerebrovascular health, laying the foundation for targeted interventions and public health strategies.

The Semmelweis-EUniWell Workplace Health Promotion Program, as part of the Semmelweis Caring University Model Program [114], targets the same occupational population being studied in the Semmelweis Study and offers a practical platform for developing interventions that address sleep-related risk factors [114]. The design and interventions of the Semmelweis-EUniWell Workplace Health Promotion Program are, in part, informed by the evidence derived from the Semmelweis Study, particularly regarding the relationships between sleep duration, shift work, and unhealthy brain and cerebrovascular aging. One critical focus of the Semmelweis-EUniWell Workplace Health Promotion Program is sleep education, aimed at increasing awareness about the importance of balanced sleep duration. Educational programs emphasize practical strategies to improve sleep hygiene, such as limiting exposure to blue light-emitting digital devices before bedtime, establishing regular sleep schedules, and creating conducive sleep environments. By addressing behavioral contributors to poor sleep, these programs can empower participants to make healthier choices that promote optimal sleep patterns. Another priority of the program involves shift work management, recognizing the role of circadian misalignment in disrupting sleep duration and quality. Organizational changes, such as implementing rotating shift schedules that align more closely with natural circadian rhythms and incorporating protected rest periods, could help mitigate the adverse health effects of shift work. These adjustments aim to reduce the biological stressors of irregular schedules, particularly among healthcare professionals such as nurses and doctors. To further address sleep disturbances exacerbated by occupational stress, the Semmelweis-EUniWell Workplace Health Promotion Program incorporates behavioral interventions. Additionally, the program explores strategies to realign circadian rhythms disrupted by shift work. Circadian alignment interventions, such as bright light therapy during wakefulness and melatonin supplementation to support sleep onset, aim to restore the body’s natural sleep–wake cycle. These approaches can alleviate the detrimental effects of circadian misalignment, which has been closely linked to cardiovascular and cerebrovascular outcomes. Recognizing that workplace stress is a major contributor to sleep disturbances, the Semmelweis-EUniWell Workplace Health Promotion Program also includes stress reduction programs. Interventions such as mindfulness training, relaxation techniques, and mental health support services are incorporated to address both psychological and physiological aspects of stress. By reducing workplace stress, these programs can positively influence sleep duration, quality, and overall well-being. Informed by the findings of the Semmelweis Study, the Semmelweis-EUniWell Workplace Health Promotion Program is well-positioned to offer evidence-based solutions for reducing stroke risk and promoting healthy aging in high-risk occupational populations. While the Semmelweis Study provides critical insights into the mechanisms linking sleep disturbances to cerebrovascular outcomes, the Semmelweis-EUniWell Workplace Health Promotion Program translates this knowledge into targeted, practical interventions. Together, these efforts address modifiable risk factors for stroke and align with broader public health goals to improve sleep health, reduce disease burden, and enhance the well-being of healthcare professionals and other vulnerable occupational groups.

Despite its strengths, this meta-analysis has certain limitations that must be considered. Most notably, the reliance on self-reported sleep duration in many of the included studies introduces the potential for recall bias and inaccuracies. Objective sleep measurements, such as actigraphy or polysomnography, would provide more precise assessments. While age and sex were considered during the literature review, the available data did not allow for meaningful subgroup analyses. Additionally, significant heterogeneity was observed, particularly in studies examining stroke incidence, reflecting variability in study design, population characteristics, and sleep assessment methods. While this heterogeneity underscores the complexity of the relationship between sleep and stroke, it also highlights the need for standardized definitions of sleep duration and cerebrovascular outcomes in future research. Evidence of publication bias, particularly in incidence studies, suggests a potential overestimation of the effect size due to selective reporting of positive findings. Although differentiating stroke subtypes (ischemic vs. hemorrhagic) would provide additional insights, most included studies did not distinguish between these subtypes. Future research should address this gap.

The findings of this meta-analysis also open important avenues for future research into the mechanisms linking sleep disturbances to stroke. Longitudinal studies integrating advanced biomarkers of accelerated aging [115–119], inflammation, endothelial dysfunction, and neurovascular health [20, 21] are needed to clarify the underlying biological pathways [120]. Furthermore, interventional studies investigating sleep-focused strategies, such as cognitive-behavioral therapies for insomnia, circadian alignment techniques, and workplace modifications for shift workers, will be essential for developing targeted approaches to reduce stroke risk.

In conclusion, this meta-analysis highlights a U-shaped relationship between sleep duration and stroke risk, with both short and long sleep durations significantly increasing the risk of stroke incidence and mortality. These findings underscore the importance of balanced sleep duration as a modifiable risk factor for cerebrovascular health. By informing the design and focus of the Semmelweis Study, this work provides a critical foundation for exploring the role of sleep disturbances and occupational factors in cerebrovascular outcomes. Addressing sleep as a modifiable risk factor is a key step in improving stroke prevention strategies, particularly in vulnerable populations such as shift workers, and advancing efforts to promote healthy aging and reduce the global burden of stroke.
